# Dual-modality impairment of implicit learning of letter-strings versus color-patterns in patients with schizophrenia

**DOI:** 10.1186/1744-9081-1-23

**Published:** 2005-12-12

**Authors:** Ming-Jang Chiu, Kristina Liu, Ming H Hsieh, Hai-Gwo Hwu

**Affiliations:** 1 Department of Neurology, National Taiwan University Hospital, College of Medicine, National Taiwan University, No. 7, Chung-Shan S. Rd., Taipei, Taiwan; 2 Department of Psychiatry, National Taiwan University Hospital, College of Medicine, National Taiwan University, No. 7, Chung-Shan S. Rd., Taipei, Taiwan

## Abstract

**Background:**

Implicit learning was reported to be intact in schizophrenia using artificial grammar learning. However, emerging evidence indicates that artificial grammar learning is not a unitary process. The authors used dual coding stimuli and schizophrenia clinical symptom dimensions to re-evaluate the effect of schizophrenia on various components of artificial grammar learning.

**Methods:**

Letter string and color pattern artificial grammar learning performances were compared between 63 schizophrenic patients and 27 comparison subjects. Four symptom dimensions derived from a Chinese Positive and Negative Symptom Scale ratings were correlated with patients' artificial grammar implicit learning performances along the two stimulus dimensions. Patients' explicit memory performances were assessed by verbal paired associates and visual reproduction subtests of the Wechsler Memory Scales Revised Version to provide a contrast to their implicit memory function.

**Results:**

Schizophrenia severely hindered color pattern artificial grammar learning while the disease affected lexical string artificial grammar learning to a lesser degree after correcting the influences from age, education and the performance of explicit memory function of both verbal and visual modalities. Both learning performances correlated significantly with the severity of patients' schizophrenic clinical symptom dimensions that reflect poor abstract thinking, disorganized thinking, and stereotyped thinking.

**Conclusion:**

The results of this study suggested that schizophrenia affects various mechanisms of artificial grammar learning differently. Implicit learning, knowledge acquisition in the absence of conscious awareness, is not entirely intact in patients with schizophrenia. Schizophrenia affects implicit learning through an impairment of the ability of making abstractions from rules and at least in part decreasing the capacity for perceptual learning.

## Background

**Figure 1:**
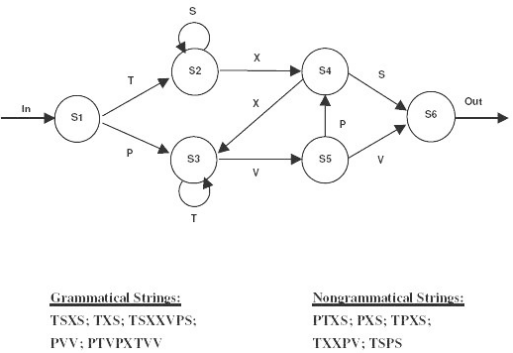
Finite-state artificial grammars used in this study are displayed in the diagram. Grammatical lexical strings are generated by traversing the legal paths connecting the notes and the arches as shown in the diagram. Non-grammatical strings are generated by concatenating the labels that violate the flow of the diagram in one place in the diagram. Examples of grammatical lexical strings and nongrammatical lexical strings are shown.

**Figure 2:**
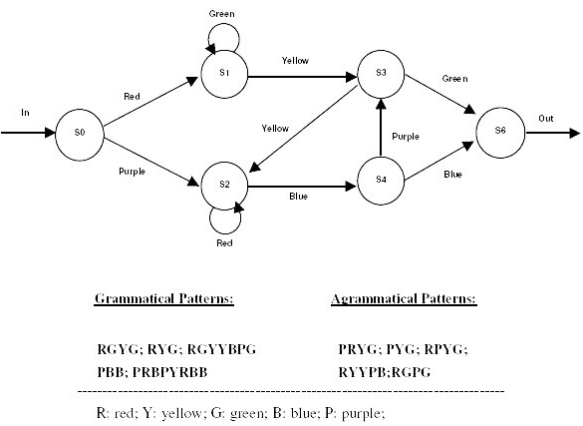
Finite-state artificial grammars used in this study are displayed in the diagram. Grammatical color patterns are generated by traversing the legal paths connecting the notes and the arches as shown in the diagram. Non-grammatical color patterns are generated by concatenating the color bars that violate the flow of the diagram in one place in the diagram. Examples of grammatical and non-grammatical color patterns are shown.

Implicit learning refers to the acquisition of embedded tacit knowledge without conscious awareness. In this form of learning, a deliberate effort to memorize or problem solve is not required. In most cases the content of knowledge gained is not immediately accessible to the knowledge holder [1,2]. For example, after repeated exposure to sequential patterns, subjects become sensitive to the statistical relationships among the adjacent elements in the stimuli [3]. Implicit learning is thought to be how children acquire their native language [4,5], how we form impressions about people, and how inferences are drawn from experiences [6-8]. Artificial grammar learning is one of the most commonly adopted approaches for implicit learning investigations. Artificial grammar learning is an implicit process, detecting the regularities in a series of stimuli generated by a finite-state-rule system (Figure 1 &2). Context free grammars resembling natural language rules were used to develop finite state machines that can be used to generate string fragments whose formations conform to the constraints of the rules. A test of artificial grammar acquisition is to judge whether letter-strings or color-bar sequences adhere to the rules at a level above chance. The goal is to identify general, parsimonious principles by which people discover the rules that regulate the sequences in the fragments. Artificial grammar learning tasks are thought to involve multiple non-conscious processes such as: the abstractionist account with gradual abstraction of rules embedded in the stimuli [9], the chunk strength account with awareness of the covariations of adjacent elements in the stimulus sequences [10], and the exemplar account with perceptual category learning [11,12]. Thought disorder is a cardinal feature of schizophrenia, in which broader definitions can encompass impaired performance on tasks such as object sorting, word association and tests of abstract thinking [13]. Artificial grammar learning research is thus relevant to the study of schizophrenia. We hypothesized that impaired abstract thinking may interfere with the rules abstraction component of artificial grammar learning. In addition, patients with schizophrenia have difficulty in processing sequential information that may reduce their awareness of the statistical relationship among the sequences in the stimuli. Finally, the impaired inhibition mechanism such as impaired pre-pulse inhibition in schizophrenia might disturb patients' perceptual learning abilities critical for artificial grammar learning [14].

**Figure 3:**
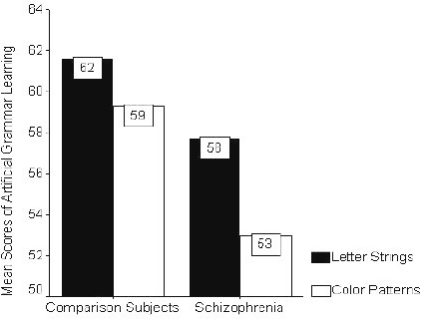
Bars represent percentages of the correct classification of grammatical exemplars by patients with schizophrenia and comparison subjects given artificial grammar learning tasks presented in letter strings and color patterns respectively. Dark color bars represent letter string condition and light color bars represent color pattern condition.

In this study, we use patients' clinical symptoms to examine how the various subcomponents of artificial grammar learning are compromised by schizophrenia. Schizophrenia symptoms have long been broadly categorized into a dichotomy of positive and negative dimensions [15]. However, results of recent statistical analyses indicated that these two dimensions were inadequate to present the full spectrum of schizophrenic syndromes [16-18]. Our previous study, using factor analysis based on 14 items of a Taiwan Chinese version of the Positive and Negative Syndrome Scale (PANSS) [19], yielded four dimensions: negative, delusion/hallucination, disorganization, and excitement whose discrimination validity were further supported by their correlations with Continuous Performance Test indices [20]. By correlating the empirically derived schizophrenia symptom dimensions with patients' artificial grammar learning of color patterns and letter strings we hope to: 1) reconcile the different findings of the intactness of artificial grammar learning in schizophrenia, and 2) examine the relationship between the sub-components of artificial grammar learning and schizophrenia symptoms.

## Methods

### Subjects

Sixty-three schizophrenic patients whose native language was Chinese were recruited from the psychiatric day hospital (Table 1). All patients were diagnosed according to the DSM-IV (Diagnostic and Statistical Manual of Mental Disorders, 4rth edition) criteria and agreed to participate in the study with informed consent as their active symptoms subsided after standard in-patient treatments. Patients had no past history of epilepsy, alcoholism, or mental retardation. None had received electro-convulsive therapy within the 3 months preceding this study. Twenty-seven comparison volunteers were recruited as the comparison group from the hospital staff and workers and matched for their ages. The comparison group's average education level was higher than the patient group's (Table 1). The study was approved by the hospital ethical committee. The clinical symptoms of the schizophrenic patients were assessed using a Taiwan Chinese PANSS [19] by the attending psychiatrists of the patients. The PANSS provided guidelines for a semi-structured interview with detailed descriptions of 7 core symptom ratings of positive and negative syndromes respectively [21,22]. Symptom scores for the four dimensions, derived from our earlier factor analysis study [20], were computed from the PANSS ratings. The four dimensions consisted of negative (blunted affect + emotional withdrawal + poor rapport + passive/apathetic social withdrawal + lack of spontaneity/flow of conversation), delusion/hallucination (delusiona1 + hallucination + suspiciousness), disorganization (conceptual disorganization + poor abstract thinking + stereotyped thinking), and excitement (excitement + hostility) factors.

**Table 1:** Demographic, clinical characteristics and neuropsychological performances of patients with schizophrenia and comparison subjects (mean ± standard deviation)

	PS	CS	
Number	63	27	
Age (years)	30.3 ± 10.1	30.0 ± 9.7	F(1, 89) = 0.017, p = 0.898
Female : Male	36:27	16:11	X^2 ^= 0.078, p = 0.683
Education (years)	12.7 ± 2.3	16.0 ± 3.0	F(1, 89) = 7.962, p = 0.006
PANSS dimensions			
Disorganization	7.3 ± 2.4		
Negative	11.6 ± 4.5		
Delusion/Hallucination	7.7 ± 3.4		
Excitement	3.2 ± 1.8		
Neuropsychological performance			
WMSR			
Verbal paired associates	22.6 ± 6.4	28.9 ± 3.0	F(3, 87) = 10.18, p < 0.001*
Visual reproduction Artificial grammar learning	59.2 ± 14.3	73.3 ± 8.4	F(3, 87) = 8.90, p < 0.001*
Lexical strings	57.7 ± 6.5	61.6 ± 5.7	F(3, 87) = 2.73, p = 0.048*
			F(5, 85) = 3.07, p = 0.014†
Color patterns	53.0 ± 6.3	59.3 ± 5.5	F(3, 87) = 8.51, p < 0.001*
			F(5, 85) = 5.48, p < 0.001†

PS: patients with schizophrenia; CS: comparison subjects; WMSR: Wechsler Memory Scales Revised; X^2^:Chi-Square test; *: MANCOVA correcting with education and age; †: correcting with education, age, verbal pared associates and visual reproduction.

**Table 2:** Spearman's rho for artificial grammar learning performances and the four PANSS symptom dimensions in patients with schizophrenia

	Disorganization	Negative	Delusion/Hallucination	Excitement
AGL				
Lexical strings	-0.225* p = 0.038	-0.188 p = 0.070	0.171 p = 0.090	0.031 p = 0.404
Color patterns	-0.248* p = 0.025	0.172 p = 0.089	-0.084 p = 0.256	-0.027 p = 0.416
WMSR				
Verbal paired associates	-0.342** p = 0.003	-2.25* p = 0.38*	0.007 p = 0.48	-0.046 p = 0.36
Visual reproduction	-0.237* p = 0.031	-0.257* p = 0.021	-0.039 p = 0.382	0.002 p = 0.494

AGL: artificial grammar learning; WMSR: Wechsler Memory Scales Revised; * p<0.05; ** p <0.01.

### Materials

The finite-state grammar employed in this study was used in a number of earlier implicit learning studies [24,25] (Figures 1 and 2). Forty-three strings and patterns of length 3~8 were generated by following the arrows in the diagrams starting from state 1 and ending at state 6. The letters used were P, S, T, V, and X. The letter strings stimuli were 24 point Times New Roman font printed in basic black against a gray background. Color patterns were generated by the same grammar with a one-to-one mapping between a letter and a color. The colors used were basic green, yellow, red, blue, and purple against a black background. Color patterns were each presented in stripes of 4 cm in height and 0.7 cm in width. The display area was about 4 cm in height and 6 cm in width on the center of the color monitor. Twenty-one of the grammatical letter strings and color patterns were used as learning stimuli. The remaining twenty-three grammatical strings and patterns were used as novel stimuli in testing. Three old items used in training were also included to make up 25 grammatical test items.

Non-grammatical strings and patterns were constructed by systematically introducing a single element violation into a grammatical string. Twenty-five letter strings and color patterns were used in each test.

Two filler tasks, one for each stimulus type, were designed to reduce the working memory effect. The letter string distracter task, consisting of four changing letter displays adjacent to one another in a 2 × 2 arrangement, was used for the letter string condition. The configurations of the letters were identical to those in the training and testing materials. The color pattern distracter task consisted of four changing color square displays adjacent to one another in a 2 × 2 fashion. The color patches were about 1 cm wide and 1 cm tall.

#### Procedures

Subjects were given the letter strings artificial grammar learning task (see Additional file 1) first followed by the color patterns learning task (see Additional file 2) after a short break between tasks. They were asked to passively observe the displays. This method, used in Reber's earlier studies [25], resulted in a higher learning effect than the more commonly used memorization task. The instructions for the training session given to the participants were purposely vague. They were only told that the experiment was some form of memory test and that they would be debriefed when the experiment was completed.

The stimulus displays were controlled by a computer program. During the training phase, the computer flashed letter strings or color patterns for each respective version of the task, regulated by artificial grammars at a rate of 500 ms per display for 7 minutes with no inter-display interval. Participants were seated approximately 60 cm from the computer monitor. A filler task lasting three minutes was inserted between the training and testing phases to minimize the working memory effect. The display time for the filler task was also 500 ms per display. During the testing phase we displayed two identical sets of fifty lexical strings or color patterns, as required by the learning task, for a total of 100 displays. These displays were shown one at a time and driven by the user's response. Subjects were asked to report if any given letter strings or color patterns looked "familiar" [26,27]. The subject's correct and incorrect responses were recorded and analyzed. Acceptances of grammatical patterns and rejections of agrammatical patterns were coded as correct responses. The same experiment protocols were followed and the same test materials were used in both groups. The real purpose of the experiment was explained to all participants during the debriefing session.

#### Statistical analysis

One-way ANOVA analysis was used to evaluate group age and education variations. A Chi-square test was used to examine the distribution by gender. Multivariate analysis of variance (MANOVA) and with a 2 (patients vs. control groups) by 2 (color patterns vs. letter strings) or (verbal versus visual memory) matrix was conducted using the general linear model procedure correcting with the covariance of education and age (MANCOVA). To further explore the effect of cognitive function such as explicit verbal and visual memory function on the implicit memory, we corrected with the covariance of verbal paired associates and visual reproduction in the WMS-R. Spearman's rho correlation analysis was performed to correlate four empirically derived schizophrenia symptom dimensions with the explicit memory function of both verbal paired associates and visual reproduction; as well as the implicit learning performances of both lexical strings and color patterns in the patient group. All analyses were performed with SPSS version 8.0 (SPSS Inc, 1998).

## Results

Although the comparison subjects and the patients were differed significantly only with their years of education we also corrected with the covariance of age to minimize the possible confounding effect. After correcting the effect of years of education and age, patients with schizophrenia exhibited poorer explicit verbal and visual memories (both p < 0.001) than the comparison subjects (Table 1). MANCOVA analysis of the implicit learning between the comparison subjects and schizophrenic patients indicated that both color-pattern and lexical-string artificial grammar learning were impaired in patients with schizophrenia. Significant group differences were found in the color pattern artificial grammar learning performance either correcting only the years of education and age (F (3, 87) = 8.51, p < 0.001) or in addition correcting the performance of explicit memory function in the neuropsychological test of both visual reproduction and verbal paired associates (F(3, 85) = 5.48, p < 0.001). While the difference in lexical string artificial grammar learning between these two groups was not as disparate as in the color pattern condition, it was just statistically significant (F (3, 87) = 2.73, p = 0.048) when we corrected only the years of education and age. We further removed the possible effect of explicit memory function of verbal paired associates and visual reproduction on the lexical-string artificial grammar learning, the results showed increased group difference (F(5, 85) = 3.07, p = 0.014). All interactions of the MANCOVA using all 2-way in repeated contrast among the age, education, verbal paired associated and visual reproduction did not reach statistical significance.

The correlation study revealed that schizophrenia has a detrimental effect on the cognitive function test. Among the four symptom dimensions, the "Disorganziation" score had adverse associations with negative correlation for both verbal paired associates (Spearman's rho, r = -0.342, p = 0.003) and visual reproduction (r = -0.237, p = 0.031). Similarly, the "Negative" symptom dimension also had significant negative correlations with both verbal paired associates (r = -0.225, p = 0.038) and visual reproduction (r = -0.257, p = 0.021). The Spearman's rho analysis on the four symptom dimensions and lexical and color pattern implicit learning in schizophrenic patients revealed that the symptom dimension "Disorganization" was negatively correlated with both lexical string (r = -0.225, p = 0.038) and color pattern (r = -0.248, p = 0.025) artificial grammar learning in patients.

## Discussion

The results of this study demonstrated that some components of artificial grammar learning were vulnerable to the pathology of schizophrenia. Patients' artificial grammar learning performances were significantly correlated with the severity of their clinical symptom dimension "Disorganization" that reflects poor abstract thinking, disorganization, and stereotyped thinking. Impaired abstract thinking in patients with schizophrenia may weaken their ability to extract the abstract rules that regulate lexical and color patterns. Patients' disorganized thoughts may reduce their sensitivity to the frequency information in the stimulus sequences. The observation favors the abstractionist account in terms of pathogenesis of the impaired artificial grammar learning. The results of our correlation study also suggested that these two underlying processes, the poor abstract thinking and disorganized thoughts, were not mutually exclusive [20]. The correlation between lexical strings and color patterns learning performances deserves attention. As mentioned earlier that learning transfer between color patterns and lexical strings in healthy adults has been reported [28]. The color patterns used in our experiments are identical to those used in that study. While a transfer study was not the goal of our study, we demonstrated that a possible common mechanism was present between these two types of stimuli. Further study is necessary to identify what this common mechanism is and whether it is responsible for learning transfer.

To what extent schizophrenia does affect perceptual priming component in artificial grammar learning? The less abnormal lexical strings learning and markedly impaired color patterns learning in patients with schizophrenia suggests perceptual priming contributed significantly to the overall performance. Lexical priming is thought to be intact in schizophrenia [29-31]. This may explain Danion et al's finding of intact lexical strings implicit learning in schizophrenia even though they took great care to avoid learning from superficial similarities in the stimuli [32]. On the other hand, we obtained but a borderline impairment of implicit memory function with lexical string learning if the performance of explicit verbal memory function was not taken into consideration. The difference would be thus pronounced after removing the confounding. This would imply that the impairment of implicit lexical string learning could be partially compensated by the explicit memory function.

The pronounced difference in the color pattern learning between patients and comparison subjects was in agreement with our previous studies [33,34]. We have proposed that the impairment in color pattern learning resulted from information overloading. Support for this argument can be found in the event related potential (ERP) P50 sensory gating deficit in schizophrenic patients [34]. We observed impaired color patterns artificial grammar learning was significantly correlated with impaired auditory P50 sensory gating while lexical strings was not. This finding suggested that faulty early pre-attention processing interfered with color pattern implicit learning. The perceptual category learning, i.e., the exemplar account, of the artificial grammar learning could thus be impaired.

Danion et al. reported intact implicit learning in patients with schizophrenia and they attributed their learning to the progressive abstraction of abstract rules [32]. They accepted that depending on the specific constraints of the stimuli, different kinds of implicit knowledge might be acquired. They constructed the test material in a way that grammatical and non-grammatical strings were similar in chunk strengths, that is, the frequency with which the adjacent elements appear in the strings. This special arrangement allowed them to rule out learning by chunk-strengths. However, their claim of intact abstract grammatical rules learning in patients with schizophrenia was not supported by the evidence from our clinical symptoms correlation study. We observed moderate to severely reduced strengths in artificial grammar learning depending on the stimulus type and found strong correlations between learning performances and abstract thinking and/or disorganized thought symptoms. We suggest the artificial grammar learning effect observed by Danion et al. may be supported by lexical priming generally agreed to be intact in schizophrenia, however, as already mentioned, confounded by the explicit memory. To what extent abstract rules abstraction is preserved in patients with schizophrenia deserves further investigation. Given the demonstrated relationship between schizophrenia symptoms and the various sub-component of artificial grammar learning we suggest that in conjunction with other neurophysiological assays such as ERP, artificial grammar learning could be regarded as an index to the neural processing.

In summary, the impaired abstract thinking may be attributed to a frontal dysfunction while the perceptual learning deficits may be related to a pre-attention sensory gating deficit which is either a temporal or a fronto-temporal mechanism. The current data does not lead us to a conclusive source of dysfunction which also might imply either a multiple mechanism of the implicit learning or it takes the cooperation of several systems or locations of the brain to complete such function. This is the major limitation of our study that we do not have comprehensive data of the cognitive function test from the comparison subjects to contrast with the patients with schizophrenia. Nor do we have data of functional images to supplement the lesion localization.

## Conclusion

In conclusion, using schizophrenic symptom dimensions and dual mode stimuli, our experimental design allowed us to partition the multi-faceted implicit learning effect. We demonstrated that the three known artificial grammar learning mechanisms: abstract rules extraction, statistical information in sequences, and perceptual priming are affected by the disease to varying degrees. Our data suggest that lexical priming in artificial grammar learning is less affected or partially preserved in schizophrenia. However, this component only accounts for a partial learning effect while other mechanisms such as abstract thinking and statistical information in sequences are disturbed by the disease.

## Lists of abbreviations

DSM-IV: Diagnostic and Statistical Manual of Mental Disorders, 4rth edition

ERPs: Event Related Potentials

MANOVA: Multivariate analysis of variance

PANSS: Positive and Negative Syndrome Scale

WMS-R: Wechsler Memory Scales Revised Version

## Competing interests

The authors declare that they do not have any competing interests.

## Authors' contributions

Ming-Jang Chiu: concept and design, acquisition of data, analysis and interpretation of data, manuscript drafting;

Kristina Liu: concept and design, acquisition of data, analysis and interpretation of data, manuscript drafting; She is of equal contribution with Ming-Jang Chiu.

Ming H. Hsieh: acquisition of data, analysis of data;

Hai-Gwo Hwu: concept and design, interpretation of data, manuscript revising;

## Supplementary Material

Additional File 1 Double clicks on 'Additional file 1' can start a program named lexical_liu to play with the letter strings explicit learning task. Please notice that the program may not be optimized for display on your computer monitor. Click here for file

Additional File 2 Double clicks on 'Additional file 2' can start a program named color_liu to play with the color patterns explicit learning task. Please notice that the program may not be optimized for display on your computer monitor. Click here for file
